# Classifying osteoarthritis status from synovial fluid using attenuated total reflectance Fourier transform infrared spectroscopy

**DOI:** 10.1016/j.ocarto.2026.100891

**Published:** 2026-11-04

**Authors:** V-P. Karjalainen, M. Mannerkorpi, S. Das Gupta, A. Turkiewicz, N. Ali, P. Önnerfjord, M. Englund, S. Saarakkala

**Affiliations:** a Research Unit of Health Sciences and Technology, Faculty of Medicine, University of Oulu, Finland; b Department of Biomedical Engineering, Lund University, Lund, Sweden; c Lund University, Faculty of Medicine, Department of Clinical Sciences Lund, Orthopaedics, Clinical Epidemiology Unit, Lund, Sweden; d Lund University, Faculty of Medicine, Department of Clinical Sciences Lund, Rheumatology and Molecular Skeletal Biology, Lund, Sweden; e Department of Diagnostic Radiology, Oulu University Hospital, Oulu, Finland

**Keywords:** Synovial fluid, Osteoarthritis, Partial least squares discriminant analysis, Attenuated total reflectance Fourier transform infrared spectroscopy, Total protein content

## Abstract

**Objective:**

We investigated attenuated total reflectance Fourier transform infrared (ATR-FTIR) spectroscopy of human synovial fluid across different osteoarthritis (OA) stages (healthy, early OA, late OA). Specifically, we evaluated partial least squares discriminant analysis (PLS-DA) performance for classifying OA status and examined associations between total protein concentration and Amide I and II absorbance peaks.

**Design:**

Synovial fluid of healthy (N = 12), early OA (N = 13), and late OA (N = 14) donors were analyzed using ATR-FTIR spectroscopy. A PLS-DA model classified the OA status. We used linear regression, adjusted for age and sex, to estimate associations between total protein content and Amide I and II absorbances. Group differences in spectral peaks were evaluated using a linear mixed model, similarly adjusted.

**Results:**

Late OA samples showed absorbance (a.u. [95% CI]) differences compared with healthy and early OA at several peaks, including 1647 cm^−1^ (0.45 [0.18,0.71]), 1452 cm^−1^ (−0.28 [−0.44,−0.13]), 1400 cm^−1^ (−0.27 [−0.39,−0.15]), 1119 cm^−1^ (−0.58 [−0.72,−0.44]), 1079 cm^−1^ (−0.25 [−0.37,−0.13]), and 1039 cm^−1^ (−0.46 [−0.61,−0.31]). The PLS-DA achieved 95% overall accuracy in classifying healthy, early OA, and late OA samples. Total protein content positively associated with Amide I and II absorbances. Amide II showed positive group-level associations in healthy (β = 12.0 [5.4,18.6]) and early OA (β = 9.3 [−0.4,19.1]), but association attenuated in late OA (β = 1.7 [−4.1,7.6]).

**Conclusions:**

ATR-FTIR spectra of synovial fluid are associated with OA status. The ability to differentiate healthy, early OA, and late OA samples demonstrates the potential of ATR-FTIR spectroscopy as a label-free method for assessing joint health without adding chemical compounds.

## 1 Introduction

The whole knee joint is affected by osteoarthritis (OA), resulting in a complex cascade of molecular changes that occur and macroscopic changes in all joint tissues, such as loss of cartilage homeostasis, extracellular matrix (ECM) degradation with proteoglycans and collagen within the affected knee tissues [1], [2], [3], [4], [5], [6]. As the joint tissues degrade, they release their molecular components, including non-coding RNAs, growth factors, other proteins, and cells into the synovial fluid [7]. As the synovial fluid connects various joint tissues at biomechanical, immunological, and molecular levels, it provides a unique biochemical window in studying joint health [7]. Synovial fluid research is crucial not only for understanding its function in OA pathogenesis but also for identifying biomarkers with potential diagnostic utility [8].

It is widely recognized that in OA molecular-level disruptions begin earlier than observed structural tissue-level changes, and even years before clinical symptoms emerge, making the early stages asymptomatic and challenging to detect [9], [10]. Multiple studies have reported differences between the biomolecular composition of early OA and late-stage OA synovial fluid [11], [12], [13]. Although several promising biomarkers have been identified from synovial fluid in human and animal studies that are associated with early and late-stage OA, their clinical applicability remains indefinite, either due limited sample number or limited evaluation of biomarker changes over time [10], [14], [15], [16]. Additionally, their relevance to early disease processes remains unclear. Therefore, identifying biomarkers that could detect multiple stages of OA could open a window for therapeutic interventions to potentially be more effective [10].

Spectroscopic analytical techniques such as nuclear magnetic resonance and attenuated total reflectance Fourier transform infrared (ATR-FTIR) spectroscopy offer a promising avenue for enhancing OA diagnostics, particularly through the analysis of synovial fluid [17], [18], and can be used parallel with conventional immunoassays, and liquid chromatography–mass spectrometry. ATR-FTIR spectroscopy offers many advantages over other analytical techniques, such as being relatively inexpensive, a low sample preparation and volume requirement, and the ability to work with aggregated samples, thanks to the ATR technique that uses samples dried on the crystal surface.

Absorbance of the ATR-FTIR spectra has been studied to increase with total protein content in spiked human serum samples [19]. As the Amide I and II bands originate from peptide-bond vibrational modes [20], their intensity generally increases with protein quantity, while their peak position and shape may also shift when protein secondary structure is altered by disease processes. As the differences in protein abundance and structure are tightly linked to the disease-related biochemical changes when comparing OA and non-OA samples [12], understanding how the total protein concentration affects the ATR-FTIR spectral features would be beneficial.

In this exploratory study, our objective was to study if ATR-FTIR could act as an overall biochemical detector of OA and differentiate between healthy, early-OA and late OA synovial fluid. To achieve this, our aim was to measure ATR-FTIR absorbance from dried human synovial fluid of deceased donors (healthy), deceased donors with mild degeneration in cartilage and meniscus (early OA), and patients undergoing total knee arthroplasty (late OA). The differences in several wavenumbers within the ATR-FTIR spectral fingerprint region (500–2000 cm^−1^), including those associated with amide I, amide II, carbohydrates, lipids, and other relevant absorbance features across the OA status groups were considered. Furthermore, the classification potential of the partial least squares discriminant analysis (PLS-DA) model into different OA status groups was assessed. Finally, we studied the group-specific association of overall protein concentration with amide I and amide II peaks of ATR-FTIR spectra.

## 2 Methods

### 2.1 Synovial fluid samples

The sample collection and analysis have been proven by the ethical review committee of Lund University (Durs: 2015/39; 2016/865; 2019/3239) and carried out in accordance with relevant guidelines and regulations by the Declaration of Helsinki principles. The synovial fluid was obtained from the knee tissue biobank MENIX, located at Skåne University Hospital in Lund, Sweden. Three different subject groups were retrieved from: i) deceased donors (hereafter called as healthy) without known clinical knee OA (as assessed through medical records and next of kin) and without macroscopic signs of degeneration, ii) deceased donors with mild joint damage without known clinical knee OA but with minor macroscopic signs of cartilage and/or meniscus degeneration in the medial compart (hereafter called as early OA), and iii) individuals with end-stage medial compartment knee OA undergoing total knee arthroplasty (hereafter called as late-stage OA). All human synovial fluids from deceased donors were retrieved within 48 h of post-mortem, centrifuged at 1800 rpm for 10 min, and preserved at −80 °C. A total of 39 synovial fluid samples from healthy (N = 12), early OA (N = 13), and late-stage OA (N = 14) were available for this study.

### 2.2 ATR-FTIR measurements

The spectra were collected using an ATR-FTIR spectrometer (Thermo Scientific Nicolet iS5, Thermo Nicolet Corporation, Madison, WI, USA), equipped with an i7-ATR diamond reflection element. Spectral acquisition was performed using the vendor-provided OMNIC software, with a spectral resolution of 4 cm^−1^ and 64 repeated scans for each acquisition. Before each measurement, a background spectrum was recorded, followed by pipetting 1 μl of synovial fluid onto the ATR crystal. After pipetting, spectral acquisition was started immediately, and 10 infrared (IR) spectra were recorded sequentially over approximately 30 min to monitor the drying process of the synovial fluid. The final spectrum (the tenth spectrum), acquired after complete drying of the sample and showing no residual water signal, was selected for subsequent analysis. To ensure consistency, the measurement was repeated three times for each sample, and the selected spectra from the dried synovial fluid were averaged. The ambient room temperature during the measurements varied from 20 to 22°C. Between measurements, the crystal was cleaned with 70% ethanol, then with Virkon, and finally with 70% ethanol again.

### 2.3 ATR-FTIR data processing

The spectra from dry synovial fluid were truncated to the fingerprint region (800–1800 cm^−1^) and were baseline-corrected using a simple offset baseline correction. A spectral shift of 5 cm^−1^ was observed between the healthy and early OA groups and the late OA group. To account for this wavenumber shift, the absorbance peak area was calculated ±5 wavenumbers around the peak position of the following wavenumbers: 1647, 1585, 1540, 1452, 1400, 1309, 1244, 1119, 1079, and 1039 cm^−1^. Assignment of the standard vibrational modes of synovial fluid are seen in Table 1 [7], [20], [21], [22], [23], [24], [25], [26].

**Table 1:** The prominent wavenumbers with their functional group assignments were identified from the healthy, early OA, and late OA spectra with IR [7], [20], [21], [22], [23], [24], [25].

Wavenumber (cm^−1^)	Assignment
1647	Amide I. C=O. C–N streching. N–H bending
1540	Amide II. C–N streching. C–C streching. N–H bending
1452	CH_3_ asymmetric bending
1400	COO− streching
1309	Amide III. C–N streching and C–H bending
1244	Amide III. SO_3_^−^ asymmetrical streching. PO^2−^ sulfate asymmetric streching
1119	C–O–S asymmetric streching. P–O–C streching
1079	C–O streching. PO^2−^ sulfate symmetric streching
1039	C–O streching

### 2.4 Multivariate (PLS-DA) analysis

Prior to the PLS-DA analysis, vector normalization was applied to remove any nonbiological variations. A three-class PLS-DA model was used to classify ATR-FTIR spectra according to disease statuses: healthy, early OA, and late OA. 39 input variables with 2076 dimensions were in the final model. Since the sample size was relatively small in this study, the model was trained and validated using a leave-one-sample-out cross-validation, testing the number of latent components from 1 to 30 to determine the optimal number of latent variables. The optimal number of latent components was found to be 20. The PLS-DA model with the highest mean value of area under the receiver operating characteristic curve (AUC-ROC) was selected. Bias was considered, and therefore, a leave-one-out CV scheme was used, which is known to be an almost unbiased estimate of the true generalization error [27]. Confusion matrix was drawn and sensitivity and specificity were calculated for each three class versus the rest of classes. Accuracy was calculated as an overall accuracy of all classes. The Matlab-code for the PLS-DA analyses, calculations of confusion matrix, sensitivity, specificity, and accuracy are available in Supplementary material.

### 2.5 Total protein content

The total protein concentration in synovial fluid samples was determined using the Pierce™ BCA Protein Assay Kit (Thermo Fisher Scientific, Rockford, IL, USA) following the manufacturer's microplate protocol.

### 2.6 Statistical analysis

Changes in IR peaks among the disease groups were analyzed using linear mixed models. The integral absorbance of each peak (i.e., area under the peak ±5 wavenumbers) was assigned as the outcome, and group and peak name and their interaction were included as fixed effects. Subsequently, we included a random effect for person and allowed heteroscedastic residuals with respect to peak. Assumptions of the model were checked using the residuals plot. The models were also adjusted for age and sex. The association between Amide I and II peaks with total protein content was analyzed with a separate linear model. The total protein content was assigned as the outcome, and the integral absorbance was set as the predictor. We included group, interaction between groups, and integral absorbance into the model to allow for different associations in each group. As this is an exploratory study, we applied no corrections for multiplicity. Inferential results are presented as estimates with 95% confidence intervals.

## 3 Results

### 3.1 Descriptives of study participants

Descriptive statistics on the study participants are presented in Table 2. All groups had few more males than female donors.

**Table 2:** Descriptive statistics on the study participants for healthy, early OA, and late OA. The study groups had similar ages and BMIs, with slightly more male donors than female ones.

Group	No. of donors	No. Female/male	Age mean (SD)	BMI mean (SD)
Healthy	12	5/7	68.8 (10.4)	26.4 (5.7)
Early OA	13	5/8	70.5 (8.5)	26.9 (5.6)
Late OA	14	6/8	71.7 (5.5)	28.1 (4.0)

### 3.2 Univariate analysis of ATR-FTIR spectra

Visual inspection of the average ATR-FTIR spectra revealed several spectral regions where the late OA group showed consistent differences compared to the healthy and early OA groups (Fig. 1). The selected peak positions (1647, 1540, 1452, 1400, 1309, 1244, 1119, 1079, and 1039 cm^−1^) correspond to well-established vibrational bands reported in the literature, primarily associated with key cartilage constituents such as collagen (amide I and II regions) and proteoglycans. All individual spectra are seen in Supplementary Fig. 1, which suggests that Late OA spectra showed less variability and more consistency in shape compared to healthy and early OA spectra. The raw, unprocessed spectral data (400–4000 cm^−1^) is found as supplementary data.

**Fig. 1:**
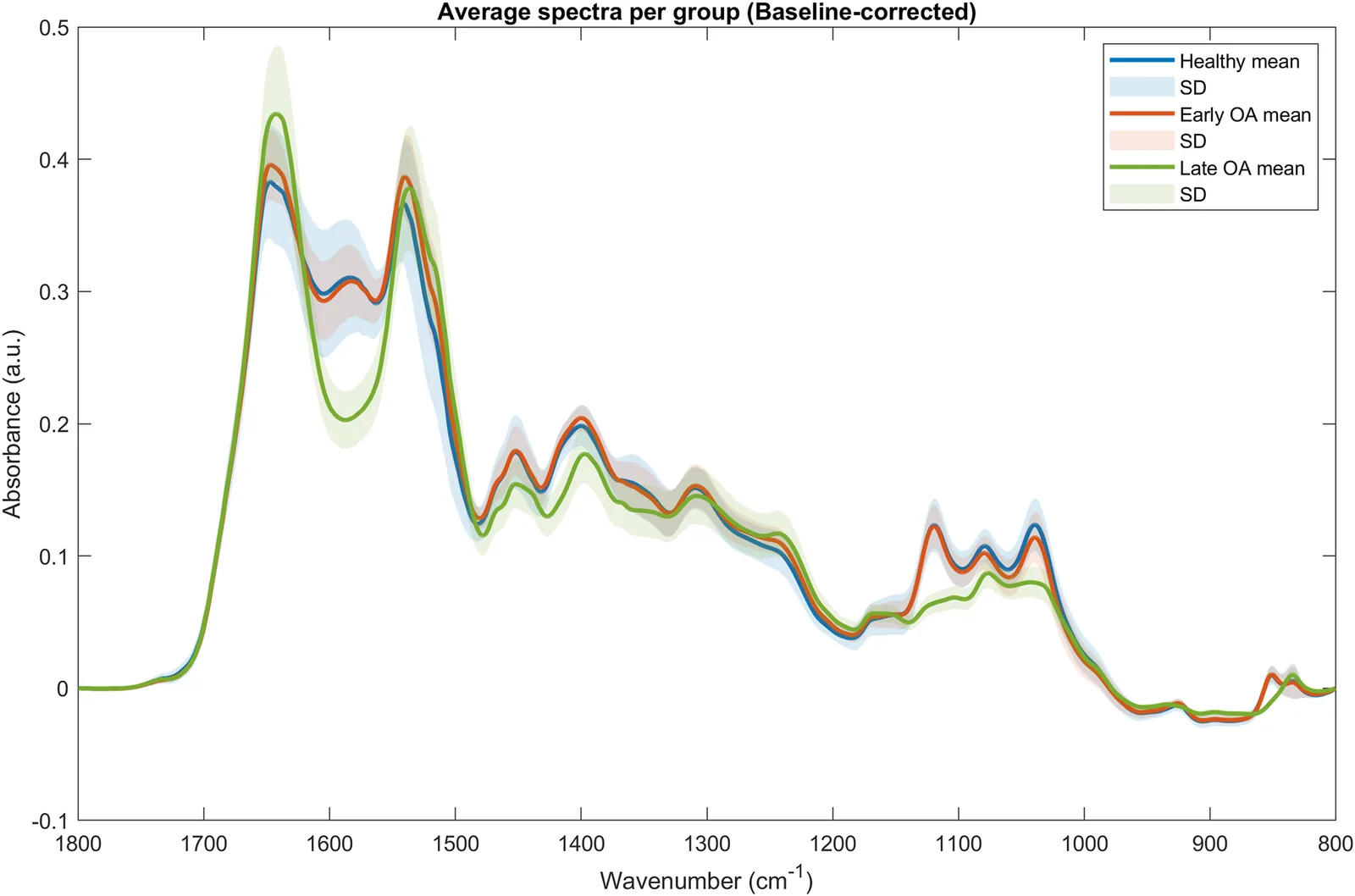
The mean absorbance ATR-FTIR baseline-corrected spectra of healthy (N = 12), early OA (N = 13), and late OA (N = 14), respectively, are shown as a function of wavenumber. The spectra are truncated to the fingerprint region of 800–1800 cm^−1^. Prominent peaks in Amide I and II regions (1647 cm^−1^, 1540 cm^−1^), bending of CH_3_ proteins and lipids (1452 cm^−1^) region, COO^−^ stretching of amino acids region (1400 cm^−1^), Amide III region (1309 cm^−1^), asymmetric stretching of PO^2−^ region and PO^2−^ sulfate asymmetric stretching (1244 cm^−1^), and the carbohydrate regions (1119 cm^−1^, 1079 cm^−1^, 1039 cm^−1^), are indicated with black arrows. a. u. = arbitrary unit.

In the Amide I region around the peak at 1647 cm^−1^, the late OA group exhibited a higher absorbance compared to both the healthy and early OA groups (Fig. 2A). In the 1585 cm^−1^ region, late OA group had the lower absorbance than healthy and early OA groups. Within the Amide II region (1540 cm^−1^), all groups yielded similar mean absorbance values (Fig. 2C). In the 1452 cm^−1^ and 1400 cm^−1^ peaks, the late OA group showed lower absorbance than both healthy and early OA groups (Fig. 2D and E). At 1309 cm^−1^ and 1244 cm^−1^ peaks, the three groups had similar absorbances with each other (Fig. 2F and G). Meanwhile, in the carbohydrate regions (1119 cm^−1^, 1079 cm^−1^, and 1039 cm^−1^), the late OA group consistently exhibited lower absorbance compared to healthy and early OA (Fig. 2H–J). The estimated differences between early OA and healthy, and between late OA and healthy groups are shown in Table 3. The estimates between early OA and late OA were essentially the same as between healthy and late OA and, therefore, are not presented separately.

**Fig. 2:**
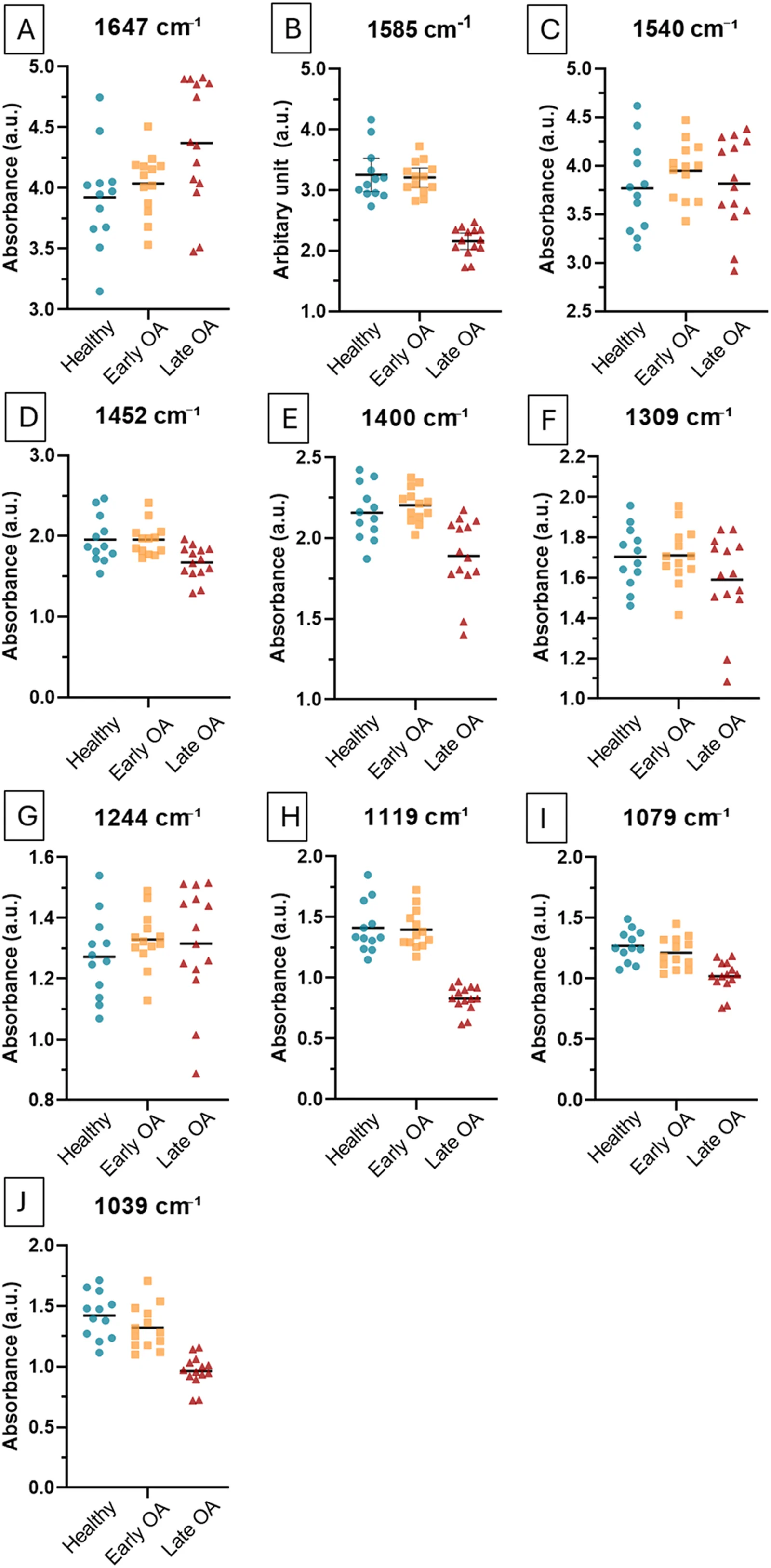
The mean integral absorbance (areas under the peaks ± 5 cm^−1^) values for prominent wavenumbers identified from the mean IR spectra of the healthy, early OA, and late OA groups. a. u. = arbitrary unit.

**Table 3:** The estimated differences for early OA vs healthy and late OA vs healthy with 95% confidence intervals (95% CI) and estimated differences after adjusting for age and sex.

Comparison	Wavenumber (cm-1)	Mean estimated differences (95% CI)		Mean estimated differences adjusted for age and sex (95% CI)	
Early OA vs healthy	1647	0.11	(-0.16.0.38)	0.13	(-0.13.0.39)
	1585	0.05	(-0.21, 0.30)	0.05	(-0.22, 0.31)
	1540	0.18	(-0.10.0.46)	0.20	(-0.06.0.46)
	1452	0.00	(-0.16.0.16)	−0.01	(-0.16.0.14)
	1400	0.05	(-0.07.0.16)	0.06	(-0.06.0.17)
	1309	0.01	(-0.12.0.13)	0.02	(-0.09.0.14)
	1244	0.06	(-0.06.0.18)	0.07	(-0.05.0.19)
	1119	−0.02	(-0.16.0.13)	−0.02	(-0.16.0.12)
	1079	−0.06	(-0.18.0.07)	−0.05	(-0.18.0.07)
	1039	−0.10	(-0.25.0.05)	−0.09	(-0.25.0.06)
Late OA vs healthy	1647	0.45	(0.18.0.71)	0.48	(0.22.0.73)
	1585	−1.10	(-0.84, −1.35)	−1.10	(-0.84, −1.36)
	1540	0.05	(-0.23.0.32)	0.09	(-0.17.0.35)
	1452	−0.28	(-0.44. −0.13)	−0.30	(-0.45. −0.15)
	1400	−0.27	(-0.39. −0.15)	−0.25	(-0.36. −0.14)
	1309	−0.11	(-0.23.0.01)	−0.10	(-0.21.0.02)
	1244	0.04	(-0.08.0.16)	0.06	(-0.06.0.18)
	1119	−0.58	(-0.72. −0.44)	−0.59	(-0.73. −0.45)
	1079	−0.25	(-0.37. −0.13)	−0.25	(-0.37. −0.12)
	1039	−0.46	(-0.61. −0.31)	−0.45	(-0.61. −0.30)

### 3.3 PLS-DA classification of ATR-FTIR spectra

The best PLS-DA classification model between the healthy, early OA, and late OA groups, when comparing the performance in terms of AUC-ROC, was obtained with 20 latent components. Chosen classification model was based on the optimization plots of AUC-ROC vs. tested latent variables (Supplementary Fig. 2) and mean squared error of prediction vs. tested latent variables (Supplementary Fig. 3). The weights of the first three latent components are shown in Supplementary Fig. 4. The variable importance in projection (VIP) scores of the model are shown in Supplementary Fig. 5 and regression coefficient vector for the model in Supplementary Fig. 6. Fig. 3 presents the confusion matrix, which illustrates the PLS-DA model's performance in correctly and incorrectly classifying the IR spectra. The sensitivities for the healthy, early OA, and late OA groups were 83 %, 100 %, and 100 %. The specificities were 100 %, 92 %, and 100 %, respectively. The overall accuracy of the model was 95 %.

**Fig. 3:**
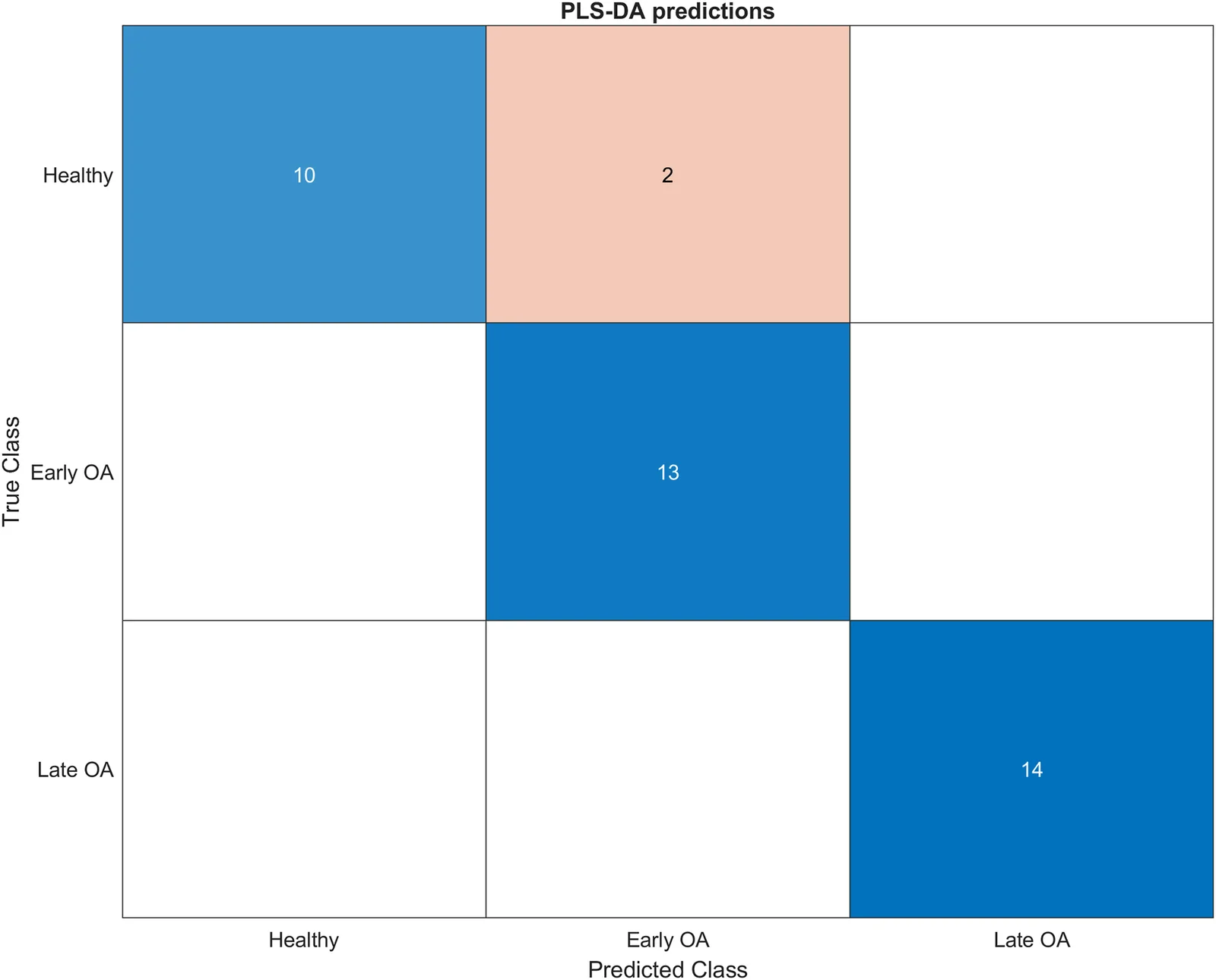
A confusion matrix illustrating the performance of the PLS-DA model across the three classes (i.e., healthy, early OA, and late OA). Each column represents the predicted class for a given group, while the diagonal elements indicate the number of correct predictions for each respective group.

### 3.4 Association of amide I and amide II peaks with total protein content

Absorbance for Amide I and Amide II peaks showed a positive association with total protein content (Fig. 4, Table 4). Similarly, Amide I and II peaks highly correlated with one another. In Amide II, a positive association with total protein content was evident in both healthy and early OA groups and attenuated in the late OA group. Similar results, but with weaker evidence of interaction with the group, were seen for Amide I.

**Fig. 4:**
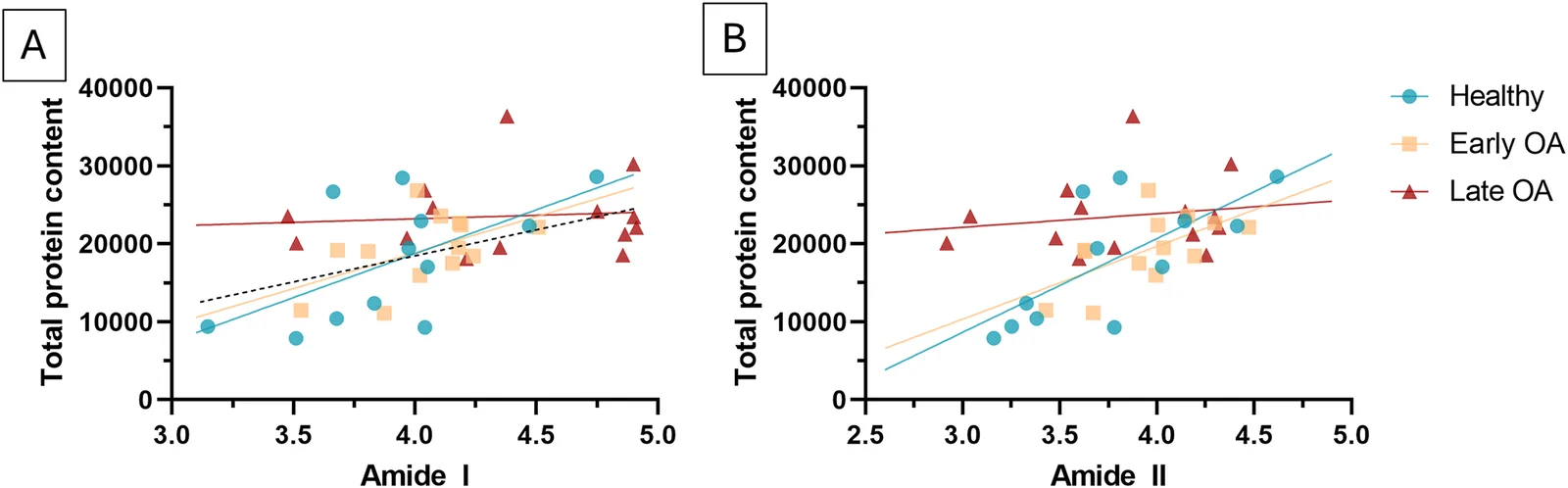
A) Association of the Amide I peak absorbance (a.u.) with total protein content for each OA status group. The black-dashed plot shows the common slope for the combined groups. B) Association of the Amide II peak with total protein content for each OA status group.

**Table 4:** Association between absorbances (a.u.) of Amide I and Amide II peaks (per 1 unit) and total protein content. The Amide I peak is estimated in one common slope, while the Amide II peak is estimated with group-specific slopes.

Peak	Group	Slope (95% CI) (x10^3^)	Slope (95% CI) adjusted for age and sex (x10^3^)
Amide I (1647 cm^−1^)	Total	5.2 (0.7.9.8)	5.3 (0.6.10.0)
Amide II (1540 cm^−1^)	Healthy	12.0 (5.4.18.6)	12.1 (5.0.19.3)
	Early OA	9.3 (−0.4.19.1)	9.6 (−1.0.20.1)
	Late OA	1.7 (−4.1.7.6)	2.4 (−4.0.8.8)

## 4 Discussion

In this explorative study, we used ATR-FTIR spectroscopy to study changes in ATR-FTIR spectra of healthy, early-stage OA, and late-stage OA human synovial fluids. Visually, late-stage OA samples could be easily differentiated from healthy and early OA groups across several spectral regions. Moreover, we trained an exploratory PLS-DA model that classified IR spectra between healthy, early OA, and late-stage OA with an overall accuracy of 95 % in a leave-one-sample-out cross-validation setup. Finally, we associated the Amide I and Amide II absorbance peaks with total protein content, which showed a positive association for both on a common level and for the healthy group on a group level for Amide II.

We found that the late-stage OA group differentiated from the healthy and early OA groups across several absorbance peaks in the fingerprint region. The synovial fluid from late OA donors showed an increase in absorbance around the amide I region compared to that from healthy and early OA donors. These spectral peaks, observed at 1647 cm^−1^, likely correspond to variations in symmetric C=O stretching, C–N stretching, and N–H bending vibrations, which have been used to quantify the collagen content from articular cartilage [28]. In addition, collagen types I and III, which have an abundance of N–H and C–N bonds, have been shown to increase in OA in synovial fluid [6], [12], [29]. Collagen type III may be upregulated during cartilage repair and fibrotic responses, indicating a role in ECM remodeling [30], [31]. However, the amide I peak is a sum of all proteins present in synovial fluid and is also influenced by their secondary structures [32], [33]. Thus, the elevated absorbance in that region may also be attributed to increased levels of several other proteins, released by surrounding joint tissues, like collagen type I^6^, matrix metalloproteinases [29], [34], and fibronectin [12], [29], which have all been reported to increase in the synovial fluid of individuals with OA and are known biomarkers and molecular mediators for OA. For instance, proteomic analysis of synovial fluid from matrix metalloproteinases are known to degrade the ECM proteins like collagen [34] and ECM degradation by enzymes causes fragmentation of fibronectin, which has been associated with inflammation in OA [5]. Therefore, elevated absorbance in the amide I region could suggest progressive ECM degradation and protein remodeling associated with OA progression.

Lower absorbance was observed in the late OA group at 1585 cm^−1^, 1452 cm^−1^, 1400 cm^−1^, 1119 cm^−1^, 1079 cm^−1^, and 1039 cm^−1^. The 1452 cm^−1^ peak has been related to collagen deformation, while the 1400 cm^−1^ band has been assigned to COO^−^ symmetric stretching and associated with carboxylate groups in glycosaminoglycans and other biomolecules [21], [35]. The 1119 cm^−1^ peak has been suggested to arise from C–O–S antisymmetric stretching [36]. The 1079 cm^−1^ peak is attributed to C–O stretching vibrations of carbohydrates in collagen and proteoglycans [22] and to PO_2_^−^ stretching modes associated with nucleic acids and phospholipids [37]. The 1039 cm^−1^ peak originates from C–O stretching vibrations in carbohydrates [20]. Differences at 1034 cm^−1^ protein peak were reported in an ATR-FTIR study of OA equine serum [38]. Some nucleic acids, like microRNAs, are dysregulated in OA and influence chondrocyte apoptosis, ECM degradation, and inflammation responses [39]. Therefore, some of the observed changes in absorbance between healthy and late OA may be attributed to the accumulation of lipids and fatty acids in the OA tissue, which have been previously linked to both pro- and anti-inflammatory processes and which have been shown to increase in the synovial fluid of OA joints [40], [41].

Overall, the carbohydrate spectral region 800-1200 cm^−1^ is particularly sensitive to hyaluronan and proteoglycans, including glycosaminoglycans (GAGs), and aggrecan, which are key components of the ECM and synovial fluids in healthy joints [42], [43]. The spectral differences observed in this region may be associated with alterations in GAG composition and structure, together with reduced concentration of hyaluronan [4], and a decrease in its molecular weight [44], both of which have been linked to OA progression. Additionally, aggrecan has been reported to fragment and deplete in late OA [12], [45]. Therefore, the observed decrease in absorbance within the carbohydrate region is most likely attributable to diminished levels of GAGs and hyaluronan. Moreover, while pure spectra of hyaluronan have been shown to also have an absorbance peak arise in the Amide I region around 1649 cm^−1^, this is likely attributed to asymmetric stretching of C=O carboxyl groups, and not Amide I vibration as similar to proteins [25]. Therefore, the effect of hyaluronic acid on Amide I peak is dismissible compared to the effect proteins have on its’ absorbance.

Interestingly, our results are consistent with a previous bovine cartilage FTIR study on collagen cross-linking [42], which showed both an increase in amide I absorbance and a decrease in sulfate and carbohydrate absorbance in the late OA group compared to early OA and controls. As the release of crosslinked peptides from collagen into synovial fluid has been shown to increase in OA, some of these spectral changes may indicate enhanced collagen cross-linking occurring in the late OA group [46].

The PLS-DA model classified healthy, early OA, and late OA synovial fluid samples with an overall accuracy of 95 % in our sample set. It is important to recognize that, since the sample size in this study was low (N = 39), the PLS model may not be generalizable outside this data set. Although using 20 latent variables resulted in the highest mean AUC-ROC and were selected as the optimal model, comparable performance was observed across a range of adjacent latent variable numbers, supporting the robustness of the classification results. The leave-one-out cross-validation was used as it provides an almost unbiased estimate of predictive performance while maximizing the training data available, which is particularly beneficial with small sample numbers [47], [48]. Existing literature lacks reporting on the classification performance of ATR-FTIR spectra from synovial fluid between human knee OA and healthy controls, highlighting the new insights provided by this study. Another study reported the classification performance of a four-class linear discriminant analysis applied to FTIR spectra of human synovial fluid from control, rheumatoid arthritis, OA, and spondylarthritis. The reported sensitivity and specificity to detect OA from these other arthritic groups were 92.0% and 100%, respectively [49]. However, there are studies where FTIR spectral classification has been conducted for animal synovial fluid samples. For instance, Malek et al. studied FTIR spectra of synovial fluid from canines and reported classification accuracies of 98.6% [15], 93.4% for short-term synovial fluid storage, and 95.8% for long-term synovial fluid storage [50], using the PLS-DA algorithm. Moreover, another study tested several classification algorithms to classify FTIR spectra of synovial fluid from canines with and without OA, reporting accuracies ranging from 93.23% to 95.95% [51]. Similarly, a previous preliminary study measured OA stage from synovial fluid with ATR-FTIR and chemometrics [52]. Unlike our study, they employed a broader fingerprint region (1700 - 450 cm^−1^) and defined the cohorts differently, using total knee replacement patients as OA group and individuals with anterior cruciate ligament injuries as non-OA control. However, the study used wider fingerprint region, but reached the overall accuracy rate of 85%, which is similar to our classification results [52]. Despite utilizing a wider spectral range, their reported overall accuracy (85%) is comparable to our classification performance. Panizzi et al. studied equine synovial fluid and reported that the random forest algorithm has an accuracy of 70–87% in detecting surgically induced OA, compared to OA control, sham, or sham control groups [53]. Our classification results are comparable to previous studies, achieving an accuracy of 95%. Some misclassification was expected as all the groups had some variation in the spectral shape. Meanwhile, classification between healthy and early OA was more challenging, as two healthy spectra were misclassified as early OA group. However, this could suggest that these deceased donors had already minor molecular changes incurring despite macroscopically labelled healthy. We suggest that for future studies, a larger number of synovial fluid samples should be analyzed to increase the generalizability of the model.

To further interpret the PLS-DA model, we examined both the VIP scores and regression coefficient vectors. The VIP analysis identified the most influential spectral regions for classification at approximately 1680, 1647, 1585, 1540, 1452, 1119, 1039, and 840 cm^−1^, while the regression coefficient vector emphasized regions around 1720, 1647, 1620, 1585, 1510, 1452, 1039, and 840 cm^−1^, with a pronounced negative region at 1680 cm^−1^. The spectral region around 840 cm^−1^ may reflect matrix-associated carbohydrate structures, as the 800–900 cm^−1^ region is commonly attributed to polysaccharide ring vibrations and glycosidic-linkage-related modes, providing information on carbohydrate structures like proteoglycans [54]. The region at 1680 cm^−1^ likely reflects the secondary protein structures of Amide I band [20]. As the VIP plot and weights of the first three latent components showed an increase in the uncertain spectral region around 1585 cm^−1^, we repeated the PLS-DA analysis after excluding the 1565-1605 cm^−1^ region. It was shown that this spectral region reduced classification accuracy from 95% to 90%, primarily due to increased confusion between Healthy and Early OA samples. This indicates that the 1585 cm^−1^ feature contributes to classification performance but is not essential for the overall differentiation capability of the model. Furthermore, the region observed near Amide I at approximately 1720 cm^−1^ is normally attributed to carbonyl stretching vibrations, originating from lipids and oxidatively modified biomolecules [20]. Collectively, these findings suggest that the classification model primarily captures OA-related alterations in protein composition, including collagen, as well as changes in ECM carbohydrates, particularly proteoglycan- and glycosaminoglycan-associated components.

The total protein content was associated with both with Amide I absorbance when all groups were included, but not on the individual group level after adjusting for age and sex. And Amide II. We found evidence of interaction, i.e. that the associations were strongest in the healthy and early OA groups and attenuated in late OA. Similarly to the Amide I, the Amide II peak corresponds to N–H bending coupled with C–N stretching, seen at 1557 cm^−1^, which is characteristic of protein structures [35]. Previously, protein content has been reported to increase absorbance in the Amide I region of ATR-FTIR spectra in a non-OA-related study [55]. The observed positive association between total protein concentration and IR absorbance suggests that variation in overall synovial fluid composition contributes to differences in spectral intensity between samples. Although carbohydrate and lipid concentrations were not measured in this study, prior work has shown that hyaluronic acid levels decrease significantly in OA [4], which aligns with the observed reduction in IR absorption associated with carbohydrate-related functional groups in late-stage OA. Additionally, earlier reports have shown that phospholipid concentrations increase in synovial fluid during advanced OA [4], [56]. These compositional changes highlight that multiple biochemical components may influence IR spectra. Therefore, future studies may benefit from accounting for additional constituents, such as carbohydrates and lipids, to better understand and potentially reduce variability in IR absorbance measurements.

Interestingly, the protein association for late OA was visibly different compared to healthy and early OA. In OA, degradation of ECM proteins leads to alterations in their secondary structure, like collagen denaturation and fragmentation, which are detectable with spectroscopic methods [30], [57], [58], [59], [60]. As Amide I and II peaks are sensitive not only to protein quantity but also to protein conformation and secondary structures, these structural changes can affect the spectral intensity from total protein content [32], [61]. This compositional complexity can weaken linear correlations between biochemical measures and spectroscopic signals [61]. Similarly, changes in proteoglycan content alters the biochemical environment and relative spectral contributions, which may indirectly affect Amide band interpretation [60], [62]. Therefore, the weakened correlation observed in our results with late OA group likely reflects disease-induced alterations in protein structure and matrix composition rather than a simple change in protein content. Our study highlights the limitation of interpreting Amide peak intensities with protein content in advanced OA tissue, which should be considered in future related studies [44], [56].

This study has important limitations. Firstly, it is important to acknowledge that this study is primarily methodological and is based on a limited sample size. Consequently, assigning each peak in the ATR-FTIR spectrum to a specific biological process with high confidence is challenging, as multiple underlying biochemical and structural factors may contribute to the observed spectral features. Second, the healthy and early OA synovial fluid samples are from postmortem donors. Postmortem changes can influence the biochemical composition of synovial fluid to some extent [63]. However, in this study, all fluid samples were collected within 48 h postmortem and processed using the same preparation protocols and freezing cycles. Previous study has shown that the impact of postmortem remains relatively low, still at 40 h, suggesting that the observed biochemical variations are unlikely to be significantly affected [63]. Moreover, the molecular origin of the 1585 cm^−1^ band in synovial fluid remains uncertain, and we therefore refrain from assigning this feature to a specific molecular component. The observed differences are reported for completeness and may warrant further investigation in future studies. Lastly, the relatively low number of samples in the different groups and the high number of latent variables may have increased model complexity and limited the generalizability of the classification model, meaning that its performance is unlikely to extend beyond this sample set and would most likely be lower in a larger, independent dataset tested with a separate test set.

In conclusion, this preliminary study suggests several differences in the ATR-FTIR spectra of synovial fluid between healthy, early OA, and late OA donors. Differentiating between healthy and OA stages from the synovial fluid using ATR-FTIR spectroscopy, combined with the PLS-DA algorithm, demonstrates promising potential as a label-free and accurate method for assessing joint health.

## Data availability

The full spectral data is available in the supplementary materials.

## Author contributions

Conception and design: SDG, NA, PÖ, ME, SS. Analysis and interpretation of the data: VPK, MM, SDG, AT. Drafting the article; VPK; MM. Critical revision of the article; All coauthors. Final approval of the article: All coauthors.

## Funding

This research has received financial support from Sigrid Juselius Foundation, The 10.13039/501100004359Swedish Research Council, Österlund Foundation, Gustaf V 80-Year Birthday Foundation, Governmental Funding of Clinical Research within National 10.13039/100018696Health Service (ALF), the 10.13039/501100007949Swedish Rheumatism Association, the 10.13039/501100006075Greta and Johan Kock Foundation, and the Foundation for 10.13039/100019769People with Movement Disability in Skåne.

This work was further supported by the 10.13039/501100003125Finnish Cultural Foundation (Grant no. 00220451), the Finnish Ministry of Education and Culture's Pilot for Doctoral Programs (Pilot project Mathematics of Sensing, Imaging and Modelling), and the 10.13039/501100020270AMBER program, a Marie Skłodowska-Curie Actions Program within the 10.13039/501100000780European Commission MSCA framework (10.13039/501100020270AMBER Grant agreement ID: 101126665). The funders had no role in study design, data collection and analysis, decision to publish, or preparation of the manuscript.

## Conflict of interest

ME declares consultancy for Grünenthal Sweden and Genascense. The other authors report no conflicts of interest.
