# Supplementary material for: Classifying osteoarthritis status from synovial fluid using attenuated total reflectance Fourier transform infrared spectroscopy
Source: Osteoarthr Cartil Open. 2026 Nov 4;8(4):100891. doi: 10.1016/j.ocarto.2026.100891 (PMC13770663; doi:10.1016/j.ocarto.2026.100891)
Supplement: Supplementary file 1 [file mmc1.docx]

**Supplementary material**

**Supplementary Figure 1.** Individual spectra from the fingerprint area (800 – 1800 cm^-1^) of each sample with baseline correction.

**Supplementary Figure 2.** Mean area under the receiver operating characteristic curve (AUC-ROC) of the classification model. Peak performance is achieved at 20 latent variables out of 30 measured latent variables.

**Supplementary Figure 3.** Leave‑one‑subject‑out (LOSO) cross‑validated mean squared error of prediction (MSEP) as a function of the number of tested latent variables. The y‑axis indicates the mean squared difference between true labels and predicted labels (lower is better).

**Supplementary Figure 4.** The weights of the first three latent components from the PLS model.

**Supplementary Figure 5.** Variable importance in projection (VIP) scores as a function of wavenumber (cm⁻¹). The dashed horizontal line indicates the threshold of VIP = 1. Wavenumbers with VIP scores above this threshold (highlighted in red) are considered important contributors to the model, while those below the threshold (shown in blue) have lower relevance.

**Supplementary Figure 6.** Regression coefficient vector as a function of wavenumber (cm⁻¹). The dashed horizontal line indicates zero, separating positive and negative contributions to the model. Positive regression coefficients represent spectral regions that contribute positively to the response variable, while negative coefficients indicate inverse relationships.
