# Supplementary material for: Classifying osteoarthritis status from synovial fluid using attenuated total reflectance Fourier transform infrared spectroscopy
Source: Osteoarthr Cartil Open. 2026 Nov 4;8(4):100891. doi: 10.1016/j.ocarto.2026.100891 (PMC13770663; doi:10.1016/j.ocarto.2026.100891)
Supplement: Supplementary file 2 [file mmc2.docx]

clear all; close all; clc;

%% Read the measurement file

excelFile = 'SupplementarySynovialFluidSpectralData.xlsx';

T = readtable(excelFile, 'ReadVariableNames', false);

C = table2cell(T);

S = strings(size(C));

for r = 1:size(C,1)

for c = 1:size(C,2)

S(r,c) = string(C{r,c});

end

end

S = replace(S, ',', '.');

M = str2double(S);

headers = M(1, 2:end);

validColsMask = ~isnan(headers) & headers >= 800 & headers <= 1800;

M = M(:, [1, find(validColsMask) + 1]);

X_axis = M(1, 2:end);

labels = M(2:end, 1);

XX_raw = M(2:end, 2:end);

[X_axis, unique_idx] = unique(X_axis, 'stable');

XX_raw = XX_raw(:, unique_idx);

valid = ~isnan(labels);

labels = labels(valid);

XX_raw = XX_raw(valid, :);

%% Baseline correction

baseline_points = [800 1800];%Change depending on data

polyDegree = 1;

baseline_idx = zeros(size(baseline_points));

for k = 1:numel(baseline_points)

[~, idx] = min(abs(X_axis - baseline_points(k)));

baseline_idx(k) = idx;

end

XXc = zeros(size(XX_raw));

for i = 1:size(XX_raw,1)

p = polyfit(X_axis(baseline_idx), XX_raw(i, baseline_idx), polyDegree);

baselineCurve = polyval(p, X_axis);

XXc(i,:) = XX_raw(i,:) - baselineCurve;

end

XX = XXc;

%% Encode groups

Healthy_idx = find(labels == 0);

Early_idx	= find(labels == 1);

Late_idx	= find(labels == 2);

group_vectors = zeros(numel(labels), 3);

group_vectors(Healthy_idx,1) = 1;

group_vectors(Early_idx,2)	= 1;

group_vectors(Late_idx,3)	= 1;

YY = group_vectors;

classLabels = {'Healthy','Early OA','Late OA'};

%% Plot baseline-corrected group means

fig_bc = figure('Color','w','Position',[100 100 1000 600]); hold on;

plot_group_means_with_shading(X_axis, XX, labels, classLabels);

title('Average spectra per group (Baseline-corrected)');

xlabel('Wavenumber (cm^{-1})'); ylabel('Absorbance (a.u.)'); box on;

legend('Location','northeast'); hold off;

%% PLS component optimization

XX_model = XX;

Number_of_PLS_comp = 30;

N	= size(YY,1);

nClasses = size(YY,2);

Accuracy_vector = zeros(1, Number_of_PLS_comp);

MSEP_vector	= zeros(1, Number_of_PLS_comp);

MeanAUC_vector = zeros(1, Number_of_PLS_comp);

[~, Class_true] = max(YY, [], 2);

figure('Color','w'); hold on;

for ncomp = 1:Number_of_PLS_comp

yfit_cv = zeros(size(YY));

for ii = 1:N

ind_train = setdiff(1:N, ii);

ind_test = ii;

[~,~,~,~,beta] = plsregress(XX_model(ind_train,:), YY(ind_train,:), ncomp);

yfit_cv(ind_test,:) = [1 XX_model(ind_test,:)] * beta;

end

[~, Class_pred] = max(yfit_cv, [], 2);

Accuracy_vector(ncomp) = mean(Class_pred == Class_true);

MSEP_vector(ncomp) = mean(mean((YY - yfit_cv).^2));

AUCs = zeros(1, nClasses);

for c = 1:nClasses

[~,~,~,AUCs(c)] = perfcurve(YY(:,c), yfit_cv(:,c), 1);

end

MeanAUC_vector(ncomp) = mean(AUCs);

bar(ncomp, Accuracy_vector(ncomp), 'b');

ylim([0 1]); xlim([0 Number_of_PLS_comp+1]);

xlabel('PLS components'); ylabel('Accuracy');

drawnow;

end

hold off;

%% AUC Figure

[bestAUC, bestAUC_ncomp] = max(MeanAUC_vector);

fig_auc = figure('Color','w','Position',[100 100 960 600]); hold on;

plot(1:Number_of_PLS_comp, MeanAUC_vector, '-o', 'LineWidth', 2);

plot(bestAUC_ncomp, bestAUC, 'o', 'MarkerSize', 10);

grid on; xlim([1 Number_of_PLS_comp]); ylim([0 1]);

xlabel('PLS components'); ylabel('Mean AUC-ROC');

title('Mean AUC-ROC vs latent variables');

hold off;

%% MSEP Figure

[bestMSEP, bestMSEP_ncomp] = min(MSEP_vector);

fig_msep = figure('Color','w','Position',[120 120 960 600]); hold on;

plot(1:Number_of_PLS_comp, MSEP_vector, '-o', 'LineWidth', 2);

grid on;

xlabel('PLS components'); ylabel('MSEP');

title('Cross-validated MSEP');

hold off;

%% Train final model

ncomp = bestAUC_ncomp;

KFold = size(YY,1);

Indices = crossvalind('Kfold', size(YY,1), KFold);

yfit_cv = zeros(size(YY));

for ii = 1:KFold

ind_train = find(Indices ~= ii);

ind_test = find(Indices == ii);

[~,~,~,~,beta] = plsregress(XX_model(ind_train,:), YY(ind_train,:), ncomp);

yfit_cv(ind_test,:) = [1 XX_model(ind_test,:)] * beta;

end

%% Confusion matrix

[~, Class_predicted] = max(yfit_cv, [], 2);

[~, Class_true]	= max(YY, [], 2);

classLabels = {'Healthy','Early OA','Late OA'};

yTrueCat = categorical(Class_true, [1 2 3], classLabels);

yPredCat = categorical(Class_predicted,[1 2 3], classLabels);

fig_cm = figure('Color','w','Position',[160 160 900 700]);

cc = confusionchart(yTrueCat, yPredCat);

cc.Title = 'PLS-DA predictions';

cc.XLabel = 'Predicted Class';

cc.YLabel = 'True Class';

cm = confusionmat(yTrueCat, yPredCat);

% Accuracy

accuracy = sum(diag(cm)) / sum(cm(:));

fprintf('Overall Accuracy: %.2f%%\n\n', accuracy*100);

%% Per-class performance metrics

numClasses = numel(classLabels);

precision	= zeros(numClasses,1);

sensitivity = zeros(numClasses,1);

specificity = zeros(numClasses,1);

f1score	= zeros(numClasses,1);

for i = 1:numClasses

TP = cm(i,i);

FP = sum(cm(:,i)) - TP;

FN = sum(cm(i,:)) - TP;

TN = sum(cm(:)) - (TP + FP + FN);

precision(i)	= TP / (TP + FP + eps);

sensitivity(i) = TP / (TP + FN + eps); % recall

specificity(i) = TN / (TN + FP + eps);

f1score(i)	= 2 * (precision(i) * sensitivity(i)) / (precision(i) + sensitivity(i) + eps);

fprintf('%s\n', classLabels{i});

fprintf(' Precision: %.2f\n', precision(i));

fprintf(' Sensitivity: %.2f\n', sensitivity(i));

fprintf(' Specificity: %.2f\n', specificity(i));

fprintf(' F1-score: %.2f\n\n', f1score(i));

end

%% Helper function for plots

function plot_group_means_with_shading(x, Xmat, labels, classLabels)

cols = [0.00 0.45 0.74;

0.85 0.33 0.10;

0.47 0.67 0.19];

legendEntries = { ...

'Healthy mean', ...

'SD', ...

'Early OA mean', ...

'SD', ...

'Late OA mean', ...

'SD'};

classes = [0 1 2];

hold on;

legendHandles = gobjects(6,1);

entryIndex = 1;

for k = 1:numel(classes)

idx = find(labels == classes(k));

if isempty(idx), continue; end

Xm = Xmat(idx, :);

mu = mean(Xm, 1, 'omitnan');

sd = std(Xm, 0, 1, 'omitnan');

upper = mu + sd;

lower = mu - sd;

% Mean line

h_mean = plot(x, mu, 'LineWidth', 2, 'Color', cols(k,:));

legendHandles(entryIndex) = h_mean;

entryIndex = entryIndex + 1;

% SD region

h_sd = fill([x fliplr(x)], [upper fliplr(lower)], cols(k,:), ...

'FaceAlpha', 0.15, 'EdgeColor', 'none');

legendHandles(entryIndex) = h_sd;

entryIndex = entryIndex + 1;

end

legend(legendHandles, legendEntries, 'Location','northeast');

set(gca, 'XDir', 'reverse');

hold off;

end
